# Electrically Insulating Plasma Polymer/ZnO Composite Films

**DOI:** 10.3390/ma12193099

**Published:** 2019-09-23

**Authors:** Ahmed Al-Jumaili, Avishek Kumar, Kateryna Bazaka, Mohan V. Jacob

**Affiliations:** 1 Electronics Materials Lab, College of Science and Engineering, James Cook University, Townsville, QLD 4811, Australia; Ahmed.Aljumaili@my.jcu.edu.au (A.A.-J.); Avishek.kumar@my.jcu.edu.au (A.K.); kateryna.bazaka@qut.edu.au (K.B.); 2 Physics Department, College of Science, Anbar University, Ramadi 31001, Iraq; 3 School of Chemistry, Physics, Mechanical Engineering, Queensland University of Technology, Brisbane, QLD 4000, Australia; 4 Research School of Electrical, Energy and Materials Engineering, The Australian National University, Canberra, ACT 2601, Australia

**Keywords:** electrically insulating coatings, polymer–nanoparticle composites, dielectric characteristics, biomaterials, renewable geranium oil, plasma-assisted technique

## Abstract

In this report, the electrical properties of plasma polymer films functionalized with ZnO nanoparticles were investigated with respect to their potential applications in biomaterials and microelectronics fields. The nanocomposite films were produced using a single-step method that combines simultaneous plasma polymerization of renewable geranium essential oil with thermal decomposition of zinc acetylacetonate Zn(acac)_2_. The input power used for the deposition of composites were 10 W and 50 W, and the resulting composite structures were abbreviated as Zn/Ge 10 W and Zn/Ge 50 W, respectively. The electrical properties of pristine polymers and Zn/polymer composite films were studied in metal–insulator–metal structures. At a quantity of ZnO of around ~1%, it was found that ZnO had a small influence on the capacitance and dielectric constants of thus-fabricated films. The dielectric constant of films with smaller-sized nanoparticles exhibited the highest value, whereas, with the increase in ZnO particle size, the dielectric constant decreases. The conductivity of the composites was calculated to be in the in the range of 10^−14^–10^−15^ Ω^−1^ m^−1^, significantly greater than that for the pristine polymer, the latter estimated to be in the range of 10^−16^–10^−17^ Ω^−1^ m^−1^.

## 1. Introduction

Recent progress in material technologies have resulted in the development of a multitude of preparation approaches and potential applications for novel polymer–nanoparticle composite films. The properties of these composite materials are often superior to that of pristine polymer films, allowing them to display greater mechanical strength, high elastic modulus, large surface areas, enhanced density, and controlled optoelectronic properties [1,2]. Among all composites, metal/plasma polymer composite films have revealed interesting optical, electrical, and biological properties [3]. These nanocomposites remarkably merge advantages of low-dimensional organic films with a great surface area of embedded nanoparticles, offering a wide range of possible applications [4,5]. A specific application highly depends on the inherent properties of the polymer and the unique surface electronic structure of the embedded nanoparticles.

In the biomedical field, the in vivo applications of electronic devices is experiencing a strong growth due to their unique diagnosis and treatment capabilities [6]. However, many of the currently used implants (designed for both short and long term usage) need to be properly isolated/protected from interacting with biofluids once they are inserted into living systems [7]. Prominent examples include an artificial cardiac pacemaker, a battery-powered device that assists the heart in maintaining a regular rhythm, and other practical devices for sensing and drug delivery functions [8]. As these implants require electrical power to operate, an insulating material is often used to prevent any electrical interference with adjacent bio-objects (e.g., muscles, bones, etc.) [9]. Furthermore, other devices are implanted in/near electrically active tissues, such as in the spine and brain [10]. Hence, proper insulating coatings are required to ensure that no electrical leaks take place from or to the device as these may interfere with proper device functioning or pose local or systemic health risks to the patient.

From the perspective of cell-surface interactions, it is often necessary to ensure adequate antimicrobial activity of the insulation coating to minimize the risk of implant-associated infections by reducing bacterial colonization and subsequent formation of active biofilms. Indeed, a diversity of opportunistic pathogens can initiate ‘implant-associated infections’, with the likelihood of the infection determined by various factors including the type of the device and other conditions of the surgical site [11]. It has been estimated that, in the United States, direct costs for healthcare-related infections ranging from US $28 billion to $45 billion in 1 year with a rise of 60% of these being connected to the use of synthetic medical devices [12].

The development of multifunctional surface coatings that reveal both insulation and antimicrobial properties could be accomplished through the use of metal/polymer nanocomposite films. In our previous study [13], a novel nanocomposite film was fabricated from ZnO nanoparticles (NPs) and renewable geranium oil using a single-step plasma-enabled approach. We demonstrated that a significant antibacterial activity could be achieved by incorporating a low concentration (~1%) of zinc oxide nanoparticles into inherently antibacterial geranium thin films [13]. In this report, we investigated the electrical properties of ZnO/geranium polymer (Zn/Ge) films with the intention to design composite coatings, which are electrically insulating and biologically active, serving as a relevant material for encapsulation of microelectronic systems and implantable devices. The electrical characteristics of such composites primarily rely on both the metal volume fraction and the chemical structure of the polymer, where the doping level of the composite is typically determined by the filling factor [14]. Therefore, the electrical characteristics of the fabricated ZnO/Ge composites were studied using percolation theories.

## 2. Experimental

### 2.1. Precursor Materials

Geranium essential oil (an oil rich in secondary plant metabolites) was purchased from Australian Botanical Products (ABP, Victoria, Australia) and used in the as-received condition. The precursor is a multi-component mixture that contains various hydrocarbon-rich components with broad spectrum antimicrobial activity (e.g., citronellol and geraniol) [15,16]. Geranium essential oil was selected as a precursor due to its high volatility at room temperature, which ensures no external heating or transporter gases is needed to carry the precursor molecules to the deposition region of the chamber. 

Zinc nanoparticles were formed from zinc acetylacetonate Zn(acac)_2_. Zn(acac)_2_ hydrate powder, which is a reasonably low-priced and commercially available Zn source. It was purchased from Sigma-Aldrich (Darmstadt, Germany) and was used without further modification. Zn(acac)_2_ compound was chosen owing to its relatively low decomposition temperature. This property, in particular, renders it suitable to be used for gas phase nanoparticle formation within the plasma polymer system without the need for any catalyst [17].

### 2.2. Material Fabrication

**Figure 1:**
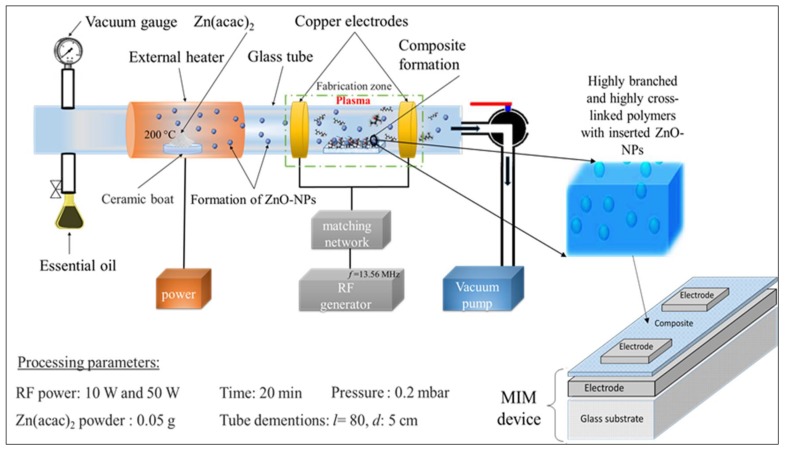
Schematic representation of the modified-plasma system used to manufacture plasma polymer/ZnO films. The metal–insulator–metal (MIM) design that was used to investigate the electrical properties of the resultant composites is also shown.

Prior to sample fabrication, substrates (typically glass slides (26 mm × 76 mm)) were cleaned and sonicated in a bath of water and commercially available decon for 20 min. Then, the substrates were washed by acetone and dried out using compressed air. Aluminum electrodes were fabricated on the top of the glass slides utilizing thermal evaporation instrument (HINDHIVAC 12A4D, Bangalore, India) under a vacuum of 7 × 10^−5^ torr. Pristine polymers and ZnO/polymer thin films were fabricated on the aluminum layer employing a modified plasma-enhanced chemical vapor deposition (M-PECVD) technique (MKS Instruments, Andover, MA, USA), as presented in Figure 1. A radio frequency, RF, signal generator (13.56 MHz) delivered power to a glass tube via a pair of external electrodes (made of copper). In the case of composite preparation, the system was modified with an external heater to achieve thermal decomposition of Zn(acac)_2_ powder (0.05 g), which was positioned inside the glass tube. Zinc oxide nanoparticles were generated in the vapor phase and incorporated within the polymer matrix as it grew. A quantity of 0.5 g of geranium oil was used in each deposition to yield a film thickness of around ~500–700 nm, with the monomer flow rate estimated to be approximately 16 cm^3^/min. Finally, another aluminum electrode was deposited on top of the result polymer films using a copper shadow mask that produced the required configuration for the metal–insulator–metal (MIM) structure, as presented in Figure 1.

All thin films were derived from geranium essential oil at input power 10 W and 50 W, so the resulting pristine polymers were termed as Ge 10 W and Ge 50 W, while the counterpart ZnO-polymers were termed as Zn/Ge 10 W and Zn/Ge 50 W, respectively.

### 2.3. Electrical Measurements

Dielectric properties of the resultant MIM devices were investigated between frequencies of 10 Hz and 100 KHz using a Hioki 3522 LCR meter (Hioki, Ueda, Japan). From estimated the thickness and area of the device, and measured C values, dielectric constant was calculated. Besides, current–voltage (I–V) measurements were conducted on the MIM structures by employing a Keithley 2636A source meter (Keithley, Cleveland, OH, USA). Data were recorded between 0 and 20 V, with steps of 0.2 V for each point, at room temperature.

## 3. Results and Discussion

**Figure 2:**
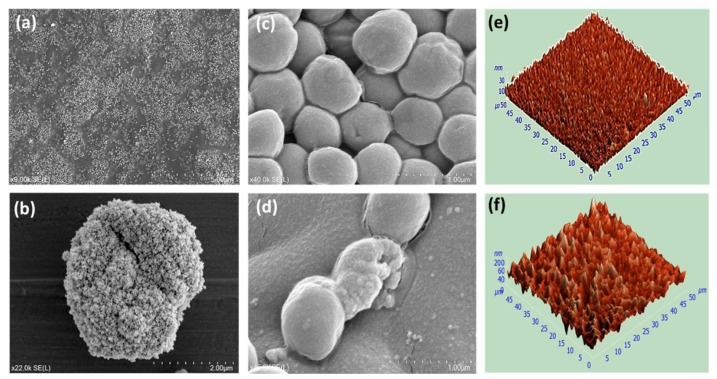
Properties of plasma polymer/ZnO composite films. (**a**) SEM image of ZnO NPs (~70–80 nm) embedded within a nanocomposite thin film acquired at ×9.0 k magnification. (**b**) SEM images of aggregation of some ZnO NPs presented in the composite acquired at ×22.0 k magnification. (**c**) and (**d**) SEM images of *S. aureus* cells cultured for 24 h (at 37 °C) on surfaces of control and Zn/Ge 10 W, acquired at ×40.0 k and 45.0 k magnification, respectively. Sterilized cover glass substrates were used as a control in all biological experiments. (**e**,**f**) Three-dimensional AFM images of pristine polymer and ZnO/composite surfaces measured at a scanning area of 50 µm × 50 µm.

Figure 2a clearly shows that ZnO NPs were formed in a ball-like structure. The average particle size was 60 nm and 80 nm for the samples fabricated at 10 W and 50 W, respectively. Furthermore, we observed some unavoidable aggregations of nanoparticles within polymers due to high cohesive energy of metals, as seen Figure 2b. These aggregations statistically represented less than 10% of the overall number of nanoparticles. Figure 2c,d show the bacterial viability of gram-negative *Staphylococcus aureus* cells when seeded on the surfaces of control (sterilized glass substrates) and composite films, respectively. It was found that around 80% of *S. aureus* cells were active on the control, while the viability of the counterpart cells were almost 31% and 42% on Zn/Ge 10 W and Zn/Ge 50 W, respectively. These results indicate a significant antibacterial activity of Zn/Ge 10 W samples owing to a combination of inherently antibacterial properties of the polymer film and the presence of ZnO NPs within the matrix of the polymer. The atomic force microscopy image (AFM) in Figure 2e shows that the pristine polymer was uniform and smooth, with an average roughness of 0.25 nm for an input power of 10 W. In contrast, composite films revealed a porous surface, as seen in Figure 2e, with a random distribution of ZnO NPs, where the average roughness was at 33.7 ± 2.1 nm for the input power of 10 W. SEM and AFM data were briefly presented in this report only to demonstrate that the material contained ZnO NPs with antimicrobial properties. A more in-depth investigation of the release of ZnO NPs and morphological, surface, chemical, and antimicrobial properties of Zn/Ge composites films can be found in our previous report [13]. 

**Figure 3:**
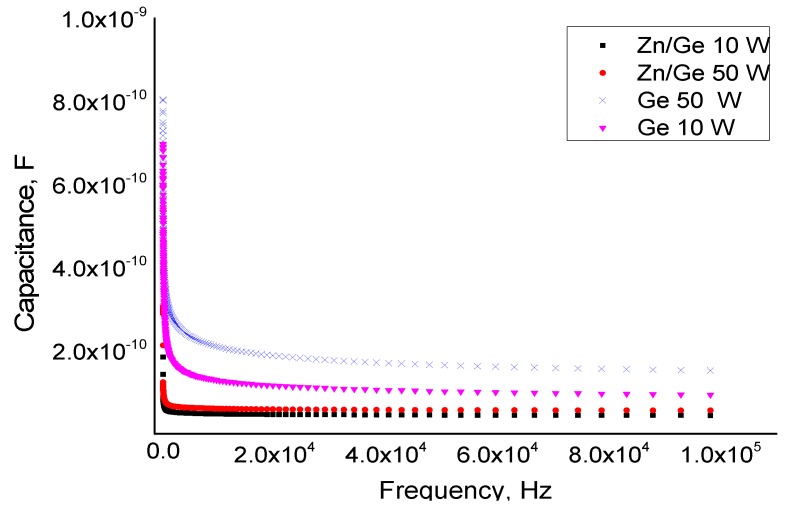
Capacitance measured for pristine and composites plasma geranium thin films fabricated at 10 W and 50 W as a function of frequency in the range of 10–100 kHz.

As the antibacterial properties were confirmed, studying the electrical properties was essential to ascertain the potential of this material as an encapsulation coating for microelectronic systems and medical implantable devices. The electric characteristics of pristine and composite polymer films were measured using capacitance measurements. The data were obtained utilizing an LCR device across a wide range of frequencies between 10 Hz to 100 KHz. In Figure 3, it can be seen that the capacitance values for pristine and composites films were approximately 10^−9^ and 10^−10^ F, which sharply decreased at low frequencies, approaching a constant value of around 10^−10^ F at high frequencies for all tested films. Regardless of frequency, presence of ZnO nanoparticles or the RF power used for film deposition had only a minor effect on the capacitance value of the pristine and composites films.

The subsequent dielectric constant of the pristine and composite thin films was calculated as a function of the given frequency and the results are presented in Table 1. For all samples, the value of dielectric constant decreased with the increase of frequency for different power of deposition. At the high frequency range (10^4^ Hz), the decreasing trend was not too sharp as compared with the lower frequency region. The decrease trend was more noticeable in ZnO/Ge composite films, since the dielectric constant inherent to ZnO nanoparticles also decreased with increasing frequencies of the applied voltage. However, no percolation behavior was noticed for the permittivity, which was observed in other studies for ZnO nanoparticles integrated within polymers [18].

SEM images showed that the ZnO nanoparticles were not ideally distributed within the polymer matrix, but rather were touching other particles creating interfaces between ZnO nanoparticles. It had been hypnotized that the interface dipole moments originate from the electrons that are confined at the ZnO/ZnO interface electronic states [18]. Indeed, the energy levels of ZnO/ZnO were dissimilar to those of ZnO/polymer interface states, where the electrons in those states respond to different frequencies. Hence, the interface dipoles related to ZnO/ZnO interfaces were possibly responsible for the variation in the dielectric constants values especially at low frequencies.

In order to theoretically estimate the contribution of the dielectric constant of pure ZnO nanoparticles to that of the composite, we used the modified Rother–Lichtenecker equation. The measured dielectric constant is given by the relation [19]:

*ε*_measured_ = exp [ln *ε*_1_ + *f*_2_(1 − *k*) ln (*ε*_2_/*ε*_1_)] (1)

where *ε*_measured_, *ε*_1_, and *ε*_2_ represent the dielectric constant of the ZnO/Ge composite, the polymer medium, and the ZnO nanoparticle, respectively, *f*_2_ represents the volume fraction of the ZnO nanoparticle, and *k* is the shape dependent factor (*k* = 0.5).

Considering the differences in the particles size (as ~60 nm particles formed at 10 W and ~80 nm formed at 50 W), the dielectric constant for ZnO NPs was evaluated at room temperature to be ԑ = 6.7 and ԑ = 6.1, respectively. It can be understood that the dielectric constant of smaller-sized nanoparticles exhibited a higher value, whereas, with an increase in particle size, the dielectric constant decreased. This is in agreement with previous findings [19]. In contrast, other studies reported that the dielectric constant increases with an increase in the size of nanoparticles [20]. The dielectric constant of pure ZnO NPs can be varied depending on the experimental conditions; for example, it was measured to be around ~10 at high frequency region (for particles size of 20 to 35 nm at 30 °C) [21]. The calculated dielectric constant in the current study could be slightly different from the real value. This discrepancy is not unusual. It is understood that ZnO has a typical metal excess defect, where oxygen is easily adsorbed on the surface of ZnO, resulting in the creation of high resistivity covers (as Schottky barriers) on the surface of the ZnO particles [22]. The bigger the ZnO particle, the smaller the ratio of surface area to particle volume. Accordingly, ZnO nanoparticle retains a larger dielectric constant. In addition, it is worth to mention that the Rother–Lichtenecker equation is valid for ideal well-dispersed particles. The non-ideal distribution of nanoparticles through the geranium polymers and the dissimilarities the particles dimension/shape could affect the accuracy of the results.

**Figure 4:**
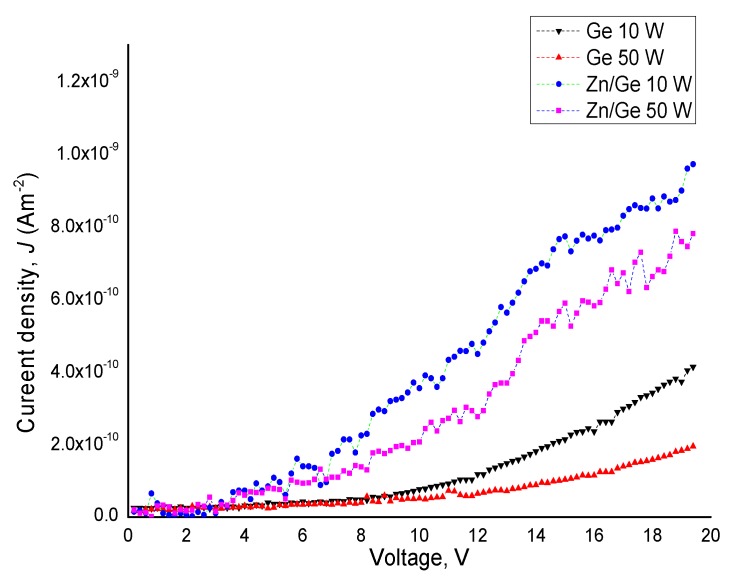
Density (J) of pristine and composite polymer films manufactured at 10 W and 50 W as a function of applied voltage (V).

Figure 4 displays the variation of current density (*J*) in the pristine and composite polymer films as a function of the applied voltage (*V*) for materials produced at 10 W and 50 W. The current density (*J*) of the films is calculated through Equation (2) [23,24]:

(2) $$J=J_{0}\exp\left(\;\frac{\beta V^{0.5}}{K_{b}Td^{0.5}\;}\;\right)$$

where, *J*_0_: the low field current density, *V*: applied voltage, *T*: absolute temperature, *k_B_*: Boltzmann’s constant, and *d*: thickness of the film. The factor *β* represents the field dropping coefficient for Richardson–Schottky (RS) conduction or Poole–Frenkel (PF) conduction mechanisms, which is given by [25] as:

(3) $$2\beta_{RS}=\beta_{PF}={\left(\frac{q^{3}}{\pi \varepsilon_{0}\varepsilon_{r}}\right)}^{1/2}$$

where, *q*: the electronic charge, ε_0_: the free space permittivity, and ε_r_: the dielectric constant. The parameters β*_PF_* and β*_RS_* are the field dropping coefficient for Poole–Frenkel and Richardson–Schottky conduction respectively. 

DC conductivity (σ) of the pristine and composited films was estimated using the current–voltage data through the following relation:

(4) σ=Jd/V

where, *d*: the thickness of the film, *J*: is the calculated current density, and *V*: the applied voltage. The conductivity for the composite films were measured in the range of 10^−14^–10^−15^ Ω^−1^ m^−1^, compared to pristine polymer films that revealed the conductivity of 10^−16^–10^−17^ Ω^−1^ m^−1^. 

In nanoparticles/polymer composites, there is a critical volume/weight concentration of fillers known as the percolation threshold. According to percolation theory, when the content of conductive filler is near the percolation threshold, the fillers connect with each other to build a continuous conducting pathway, providing the potential for electrons/carriers to transport among the fillers [26]. Thus, the composite always reveals a rapid increase in electrical properties [27]. The percolation threshold is determined by the filler shape, size distribution, interlayer thickness, temperature, physicochemical properties, and applied external field [28]. Yet, the correlation between the filler concentration and conductivity of the composite is not fully understood [29]. 

The conductivity (σ) near the percolation threshold (*ϕ*_c_) can be given by the following power law:

(5) $$\sigma_{c}=\sigma_{f}{\left(\emptyset_{f}-\emptyset_{c}\right)}^{t}$$

where, *σ*_c_: electrical conductivity of the composites, *σ*_f_: the conductivity of the filler, *ϕ*_f_: the volume portion of the filler, *ϕ*_c_: the percolation concentration, and *t*: the critical exponent (a parameter determining the power of the conductivity based on *ϕ*_c_). The critical exponent *t* depends on the dimension of the tested system, and is set between 1.6 and 2 for the three-dimensional structure. The value of $\emptyset_{c}$ was adjusted until the best linear fit was achieved in log σ vs. log σ ($\emptyset_{f}-\emptyset_{c}$). The range of critical exponent values fitted from experimental data achieved by different studies indicate that the *t* is not universal, as it varied in the range of 0.9 to 2 [30,31,32].

Based on the percolation threshold equation, we estimated the percolation threshold of Zn/Ge composites to be ~2.67%. It is clear that the Zn/Ge composites did not reach the percolation threshold since the conductivity was kept at relatively low values (10^−14^ Ω^−1^ m^−1^), rather than increasing rapidly with the addition of the particles. The relative increase in conductivity after introducing ZnO NPs could be linked to the increase in the number of dipoles, where the reformation of trap structure is induced by ZnO nanoparticles. This suggests that the formed composites do not follow the behavior of such structures as 2D or 3D conducting particles, but show more complex charge tunneling transport mechanisms that govern their conductivity [33]. The electrical conduction could also increase due to the electronic and impurity contributions arising from the zinc precursor during the thermal breakdown of zinc acetylacetonate (Zn(acac)_2_).

Some studies showed that the percolation threshold for conductivity of the ZnO/polymer system to be 15 wt.% of the polymer volume fraction [34]. Different researchers found the percolation concentration to be 2.8 vol% (ZnO = 200 nm) [35], and 0.05% for ZnO nano-rods (d = 400 nm and L = 2 µm) [36].

**Figure 5:**
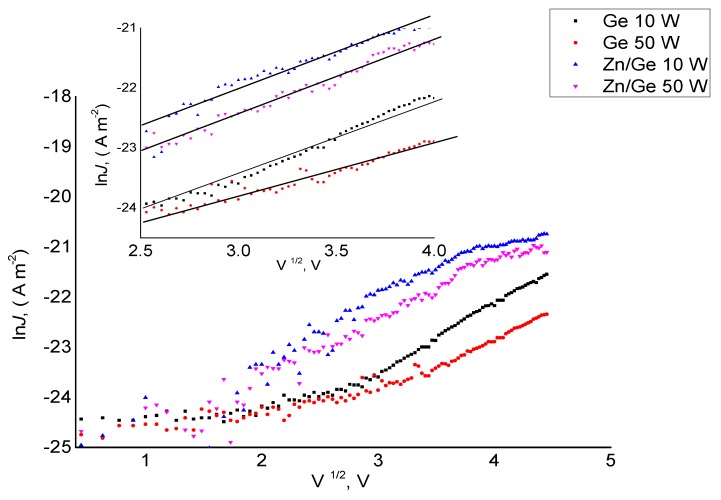
Density ln*J* with the square root of applied voltage for pristine and composite polymer films manufactured at 10 W and 50 W.

**Figure 6:**
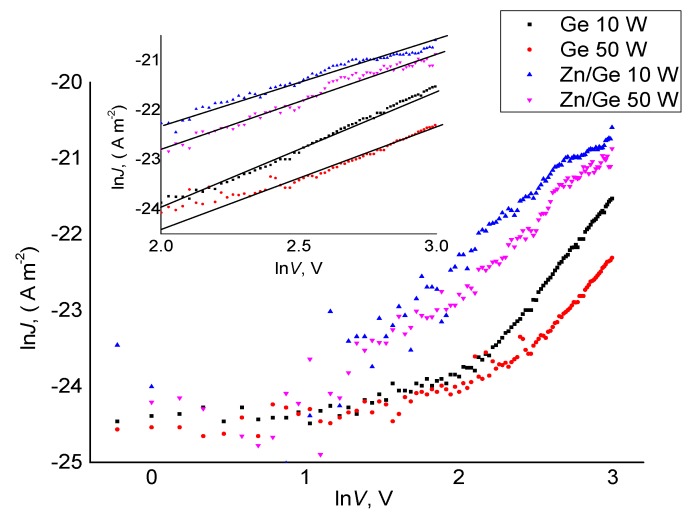
Density ln*J* with ln*V* for pristine and composite polymer films manufactured at 10 W and 50 W.

As shown in Figure 5 and Figure 6, the fitting result of ln(*J*)–(*V*^1/2^) and ln(*J*)–ln(*V*) indicates that the conduction mechanism in the high voltage range could be related to the Richardson–Schottky (RS) or Poole–Frenkel (PF) conduction. Furthermore, the fitting of ln*J* vs. ln*V* for all manufactured polymer and composites demonstrates a similar behavior as those in ln*J* vs. *V*^1/2^. By observing power law index rates and the linear fitting of the J–V plots for pristine polymer that was previously reported in [37], the Schottky mechanism could specifically dominate the charge transport of geranium polymers in the higher field region. It suggests that the ZnO NPs did not significantly affect the transport at high fields, but may indirectly change the carrier transport properties at lower fields. This outcome is expected since the ZnO NPs incorporated within the matrix were at low concentration (below the percolation threshold).

These results provide a potential pathway to adjust the bulk and surface properties of other plasma polymers that have previously been shown to display antibacterial activity [38,39,40,41] or attractive optoelectronic properties [42,43,44,45]. For example, plasma polymerized γ-terpinene thin films have shown sufficient optical transparency and photostability to be used for the encapsulation of PCPDTBT: PC_70_BM solar cells to prevent loss of efficiency [46,47], whereas polymers derived from terpinen-4-ol and linalool have been proposed as insulating interlayers in flexible electronic devices [48,49]. It can also potentially provide the means for in situ functionalization of vertically aligned graphene networks that have been fabricated from essential oils using the same plasma set-up [50,51] to improve their properties for such applications as sensing and energy storage. Indeed, there is a large range of materials that can be fabricated using plasma-enabled techniques [52,53,54], with an equally broad range of potential applications spanning medicine, electronics, energy and other fields [55,56,57,58]. 

## 4. Conclusions 

The electrical properties of pristine and ZnO doped plasma polymerized geranium oil-derived thin films were systematically investigated across the frequency range of 10 Hz to 100 KHz using metal–insulator–metal structures. The dielectric constant value was found to diminish with increasing RF input power for all samples. Irrespective of the RF power, the studied samples had almost the same frequency dependence on the dielectric constant, as it quickly declined within the low frequency zone. In addition, the mechanism of charge transport was examined via the typical current−voltage approach, which showed that the Schottky mechanism possibly dominants the charge transport in the higher field region. The resultant material demonstrated a moderately low conductivity value (10^−16^–10^−17^ Ω^−1^ m^−1^), establishing characteristics of a classical insulator. Incorporation of ZnO nanoparticles into the geranium polymer thin films did not change the nature of charge transport, as the nanocomposite films still behave as an insulator. The aforementioned properties, in addition to the antibacterial activities and other valuable features of Zn/Ge thin films (e.g., low density, relatively strong adhesion to substrates, and the high optical energy gap) introduce them as an appropriate candidate for various dielectric needs in innovative microelectronics.

## Figures and Tables

**Table 1:** The dielectric constants of pristine and composite polymer coatings manufactured at 10 W and 50 W.

Frequency (Hz)	Dielectric Constant			
	10 W	50 W	Zn/Ge 10 W	Zn/Ge 50 W
10	5.75	4.74	4.12	4.04
100	4.73	3.38	3.57	3.87
500	4.48	3.03	3.42	3.72
1000	4.4	2.93	3.37	2.64
10,000	4.17	2.66	2.06	2.35
50,000	3.69	2.37	2.98	2.73
100,000	3.05	2.04	2.78	2.42
